# L2 Arabic learners’ processing of Arabic garden-path sentences: a consistent reading pattern

**DOI:** 10.3389/fpsyg.2024.1333112

**Published:** 2024-03-08

**Authors:** Abdullah M. Seraye Alseraye

**Affiliations:** Department of Curriculum & Instruction, School of Education, King Saud University, Riyadh, Saudi Arabia

**Keywords:** garden-path structure, Arabic short vowels, past experience, heterophonic-homographic initial, L2 Arabic learners, reading comprehension

## Abstract

**Purpose:**

The main purpose of this study was to investigate to what extent the L2 Arabic learners’ reading process is affected by the incomplete representation of speech (the absence of short vowels and diacritics) while reading ambiguous sentences (garden path sentences).

**Method:**

With a self-paced reading software program, 41 non-native male students, aged from 22 to 26, enrolled in King Saud University, participated in reading 44 sentences (followed by reading comprehension questions) representing three reading conditions, plain, vowelized-discretized, and wrongly-vowelized.

**Results:**

For the reading times data, the analysis revealed that the GP structure had a significant effect on the reading processes of L2 Arabic learners; it took them longer to read the GP sentences than their non-GP counterparts. For the reading comprehension, the analysis did not reveal any significant differences between the means for the percentages of correct responses. For the comparison between the three reading conditions, a significant difference was found: it took the participants on average less time to read the GP sentences when presented plain, and more time with the incorrect representation. However, their reading comprehension was not affected.

**Conclusion:**

In addition to the good-enough model and the nature of Arabic morphology, the reading experience, is a good candidate to start with as an important factor in the interpretation of the ineffectiveness of the GP structure on the reading comprehension process of Arabic readers, in which the segregability of Arabic writing system prepare the readers to emphasize some sensory inputs and ignore others based on their past reading experience.

## Introduction

Languages are intrinsically susceptible to structural ambiguity. Indeed, “at any given point in a sentence, the available information can be ambiguous at many levels,” because languages are “structured at multiple levels simultaneously, including lexical, phonological, morphological, syntactic, and text or discourse levels” (MacDonald et al., 1994, p. 676). This structural ambiguity can result from either an optional, controlled cause or a non-optional, compulsory cause. Some researchers view the Arabic writing system as being by nature ambiguous due to the consonantal representation of its orthography (De Francis, 1989). However, structural ambiguity in Arabic is not omnipresent and internally structured but situational because of its optional and segregable representation of short vowels and diacritics.

In fact, integrating the necessary short vowels and diacritics with the consonants would turn Arabic print into a transparent orthography and should therefore disambiguate any potential structural ambiguity in the sentence by leaving only one acceptable reading for each word. Conversely, the absence of the necessary short vowels and diacritics from the consonantal representation in Arabic print would turn its orthography into a deep orthography, which means highly ambiguous orthography. An extreme example of deep orthography is a sentence that begins with a heterophonic homographic word (henceforth: HP-HG); this becomes much worse when the disambiguating region of the sentence is far from its initial word, which would very likely give rise to the garden-path phenomenon (GP) (Seraye, 2004; Alseraye, 2022). This farness in distance from the disambiguating region has been found to be from three to five words distance (Seraye, 2004, 2016; Alseraye, 2022).

**DIAGRAM 1:**
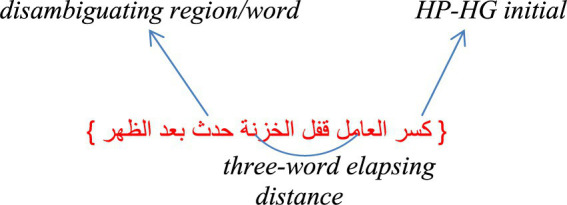
Examples of GP sentences: HP-HG initials, disambiguating word/region, and elapsed words.

In the modern Arabic language, the order of the words in a sentence is flexible, and it may take, based on stylistic variations, either one of the following word orders: VSO (i.e., verb, subject, object), SVO, VOS, and OVS (Mohammad, 2000), with no syntactic preference for one order type over the others. On the surface, this first word of the sentence could be a verb phrase (VP), as in “*,”كَسَرَ العامِلُ قفل الخزنة*” a noun phrase (NP) as in “*العامل كسر قفل الخزنة*,” or a prepositional phrase (PP), as in “في الخزنة مال” In these word orders, the ambiguity is expected with a sentence order that begins with a VP that has a third person singular verb in the past tense with enough words go in between the initial HP-HG word and its disambiguating region. For an illustration, see Diagram 1.

_____________________________________________________

In the following, a descriptive syntactic analysis of a GP Arabic sentence is laid out:

كسر العامل قفل الخزنة حدث بعد الظهر [كَسْرُ العاملِ [1] …؛ كَسَرَ العاملُ

Reading I:

- *The worker’s breaking of the lock of the safe [occurred] in the afternoon* (grammatically correct).

(a) Arabic word order: breaking + the worker + the lock + of the safe + occurred + in + the afternoon.

Correct structure: S [NP [NP, NP, PP[P, NP]], VP[PP[NP]]

(b) English word order: the worker’s breaking of the lock of the safe occurred in the afternoon.

Reading 2:

- *{broke} the worker the lock of the safe [occurred] in the afternoon* (grammatically incorrect).

(a) Arabic word order: broke + the worker + the lock + of the safe + occurred + in + the afternoon.

Mistaken structure: S [VP [NP, NP, PP [P, NP]], VP [PP [P, NP]].

(b) English word order: the worker broke the lock of the safe occurred in the afternoon (Alseraye, 2022).

____________________________________________________.

This HP-HG VP has numerous forms of the same verb that still share the same semantic root but also reflect the following different case roles in the sentence: an active verb, a gerund, and, to some degree, a passive verb in cases where the writer/speaker wants the importance or focus to be shifted toward who/what experiences the action. However, there are three conditions in which the structural ambiguity would, at its high peak, lead readers astray by garden pathing them. The first condition is when the initial word of the sentence is an HP-HG word that has numerous forms, while for the second the initial word is presented as plain, such as without the right short vowels and diacritics (i.e., unvowelized and undiacritized). For the third condition, an adequate distance elapses between the initial HP-HG word and its disambiguating region to prevent the eye from visualizing the subject and the predicate simultaneously. Therefore, these two essential parts of the sentence are not in the reader’s peripheral vision simultaneously (Diagram 1).

In the second condition, the distance that elapses between the initial HP-HG word of the sentence and its region of disambiguation is enough [five words with skilled adult readers, such as in Alseraye (2004) study; three words with the children who are beginning readers, as in Alseraye (2022) study]. This is because it helps to prevent the parser from benefiting from the linguistic context; that is, from the neighboring words that would help the parser, to a large extent, to recognize the correct form, build the right “structure tree,” and hence, avoid the disturbance expected at the region of disambiguation. Using eye movement terminology, this occurs when the reader’s eye does not capture, at one fixation, both essential parts of the sentence, the subject, and its predicate, within one visual span.

Even using a clear example of an ambiguous sentence (a passive sentence where the initial word is a verb in the passive voice), figuring out whether the verb is active or passive can sometimes be achieved without needing to reach the region of disambiguation to get it right, and without the reader being garden pathed. This can be figured out using different sources, such as the immediate previous context, the text discourse, and the reader’s experience exemplified in word and syntactic structure frequencies. Indeed, we would not expect to find such a great distance elapsing between an initial verb in the passive voice and its subject that this would garden path the reader, as are exemplified in the sentences used in Roman et al. (1985) and Hermena et al. (2015) studies. The exceptions occur in artificially created sentences. According to Ots (2021), the speaker, in the linguistic encoding stage in language production, would “assign the syntactic functions [that] are appropriate for the message and order the constituents, given the discourse and grammatical constraints” (p. 2). Similarly, the writer, I assume, would be forced to comply with these constraints, in addition to the cognitive constraints and limitations, to avoid the long distance between the HP-HG word and its disambiguating region in the sentence. The two sentences below illustrate the GP sentence as used by Hermena et al. (2015) (labeled “1”) and the modification of the same sentence that a writer is expected to make to avoid the ambiguity of the passive structure (labeled “2”). There is also a basic naturally constructed GP sentence (labeled “3”):

_____________________________________________________.

**1. سمع صرخة مدوية حينما دفعت1 الطالبة التي كانت في طريقها إلى المعمل بيدي2 زميلتها3 فهوت م**

Translation: “*Everyone heard a loud scream when the student pushed (or was pushed) on her way to the lab (by the hand of) her colleague so she fell unconscious.*”

2. سمع الجميع صرخة مدوية حينما تَمَّ دفع1 الطالبة التي كانت في طريقها إلى المعمل بيدي2 زميلتها3

3. كتب محمد منسية في مدرسته.

Translation: *Mohammed’s books were forgotten/left out in his schools.*

_____________________________________________________.

The structure of the clause in sentence 1 includes a passive voice, and without the supplement of the correct short vowels and diacritics the readers would be garden pathed. They would, as demonstrated by previous studies (Al-Fahid, 2000; Hermena et al., 2015; Seraye, 2004, 2016; Alseraye, 2022), read the HP-HG verb “دفع” as an active verb “(she) pushed: دَفَعَتْ” Then, when they reached the disambiguating region, such as the word “by the hand of” بيدي they would notice that their decisions were wrong, because they did not read the verb “دفعت” as being in the passive voice (seven words elapsing). However, in practical terms, it is expected that when writers express the meaning of such a sentence or clause they would not generally include a clause with such an ambiguous passive voice in a sentence that is so complex. They would avoid this by converting the verb “دفعت” into a phrase that says the same thing without any need to refer to an explicit subject. At the same time, they can eliminate the ambiguity without having to vowelize and diacritize the verb, by using the phrase “تم دفعها” Another strategy to avoid sentence ambiguity is to use a syntactic mechanism called topicalization, in which the focus is brought to the front, and the order of items, such as the constituents, the phrases, and its adjuncts, changed so they are close to the main verb.

The example of the GP sentences (number 3) used in Alseraye (2022) is, to some degree, representative of the type of sentences that Arab children might see in a text that is realistic and naturally structured. However, Arab readers were nevertheless garden pathed, unless they were able to guess (see Appendix A).

In addition to the current study, previous studies (Seraye, 2004; Alseraye, 2022) have already examined the behaviors of the Arab readers, both beginners (the children) and experienced (the adults), when they read. The studies have found that Arab readers who read ambiguous sentences, such as the GP ones that were embedded in short texts for reading aloud, demonstrated reading behaviors that were essentially consistent among all participants regardless of their reading skills and reading-equivalent ages (i.e., experience). Those reading behaviors are as follows:

– Very frequently, when the basic verb is embedded in a discourse, such as a text, it does matter whether the verb was correctly vowelized and diacriticized. The Arabic learners will read it as an active voice. To illustrate, the participants read the HP-HG verbs in the passive voice as “أعلن was announced” and “سمعت was heard,” as/أَعْلَنَ [he] announced / and / سَمَعَتْ [she] heard/ (i.e., active voice form), although the linguistic context would force them to read the two verbs as passive voice forms.
– The verbs in the passive voice, “اُشْتُهِرت was known” and “اُرْتُكبت was committed,” were read as active voice forms, although they were provided with the right short vowels and diacritics that would make them non-homographic words.
– Although the participants paused and hesitated over some of the HP-HG words, they were garden pathed and very frequently made no regression (reanalysis) by going back to their initial decisions and choosing the right forms of the HP-HG verbs. Indeed, some of the participants would not even pause over the HP-HP words.
– Some participants, once they realized that the forms were passive, applied the knowledge they had acquired to the verbs that followed, by initially considering them as passive forms. That is, their previous experience with the first passive verb they encounter acts as a prime and forces them to read the first verb that follows as a passive one. However, when the participants found out that they had been garden pathed, some of them made exclamations such as “لا!” meaning “No!”
– Some of the participants insisted on using the active voice form for the vowelized/diacriticized passive voice “اُرْتُكبت” pausing over the verb, saying it as an active form, although it was represented as a passive voice form.
– When the HP-HG verb, in its plain condition, had no previous context to force the reader to read it as passive rather than active, it was read as an active form. Indeed, even when the basic verb was provided with the right short vowels and diacritics that, if assembled with the consonants, would be read as a gerund, participants read it as being active, with the short vowels and diacritics ignored, and were thus garden pathed.
– An automatic attempt was made to convert a verbal noun or a gerund (e.g., استئناف appealing) into an active basic verb (استأنف [he] appealed).

In conclusion, our study, based on the post critierain assessment (reading aloud task) found the same phenomenon among the learners of Arabic as a second language (L2) who are at the advanced level and qualified to enroll in academic programs where the language of communication is Arabic as was observed within native Arab readers, both children and adults.

Therefore, we can conclude as Seraye (2004) has already stated that “it seems that the initial sentence default, to use the notions of the symbolic and associative theories of cognition (Marcus et al., 1995), was the verb and not the noun or the preposition which Arabic allows. Further, this default was characterized by the fact that it was always regarding an active-voice verb, and this was noticed even in an embedded clause when the sentence led the reader logically to a passive voice more than to an active voice” (pp. 135–136).

In the literature, the ambiguity associated with the GP structure drew the attention of researchers, who set up suitable apparatuses to answer the following two questions: “[How do] people cope with rampant ambiguity, especially syntactic ambiguity, as the linguistic signal unfolds over time? [H]ow is sentence interpretation affected by variations in syntactic complexity?” (MacDonald and Hsiao, 2018, p. 173; Alseraye, 2022).

In the Arabic literature, research has focused, to a large extent, on the text and word levels, and very few studies have addressed the structural ambiguity, particularly the GP sentences, that is caused by the absence of short vowels and diacritics (for an overview, see Roman et al., 1985 [in French, and cited in Hermena et al. (2015)]; (Seraye, 2004, 2016; Ibrahim, 2013; Taha, 2016; Saiegh-Haddad, 2017; Abu Rabia, 2019; Hermena and Reichle, 2020; Alseraye, 2022)).

In the syntactic ambiguity that has been addressed recently is ambiguity that is caused by the GP structure in Arabic and results from the optional, segregable nature of Arabic orthography. The current survey of the studies conducted in Arabic shows that very few that have been published in the literature have addressed the processing of structural ambiguity caused by the GP structure and its effect on reading accuracy and comprehension.

A study by Abu-Rabia (1995) claimed that Arabic readers can not read or comprehend sentences that were not properly vowelized unless they reanalyzed their first readings of the initial HP-HG head of the ambiguous sentence, In response to this assumption, Seraye (2004) conducted a second experiment that assessed, among other variables, the effect of the GP structure on the reading processes, reading times, and comprehension of highly skilled Arab adults (n = 35, in the 26–40 age range), in correlation with the presence and absence of correct short vowels and diacritics.

Using a self-paced reading, controlled by a moving window software program in which the sentence is read word-by-word without regression, Seraye found that the Arab adults’ reading times of the GP sentences were affected (*p* = 0.016), but that their reading comprehension was not (*p* = 0.053). The average length of time to read the two types of sentences was longer for the GP sentences (*M* = 6,747.14 ms) than for the non-GP ones (*M* = 6,259.30 ms), but their reading comprehension performance was very good on both types of sentences (*M* = 0.89, for the GP sentences, and *M* = 0.83, for the non-GP sentences, *SD* = 0.08). Therefore, from a descriptive perspective, the mean values indicate a positive relationship between the reading time length of the GP/non-GP plain sentences and the reading comprehension performance.

In addition, Alseraye’s (2004) third experiment on word naming using the E-Prime software program found that reading latency was “positively correlated with the gradual increase of the number of short vowels and diacritics” in comparison to the consonants (p. 214).

This ineffectiveness of the GP structure regarding the reading comprehension of Arab adults was attributed to two features of Arabic morphology: the core semantic element, based on the *trilateral/quadrilateral*-root, that is shared among all activated forms of HP-HG word, and the form/pattern of the Arabic word, its skeletal tier/word pattern/binyan (McCarthy, 1979, 1981), which would narrow the possible readings of the HP-HG word (Seraye, 2004). In fact, as proposed by Seraye (2004), “the predictability/productivity of word forms/patterns, affixation, etc., compensate for the lack of short vowels and diacritics in print” (p. 259).

This advantage of Arabic morphology roots and word patterns in the Arabic reading process has been, consolidated, and theoretically grounded (Seraye, 2004, 2016; Mahfoudhi, 2007; Mahfoudhi et al., 2010; Abu-Rabia, 2012; Abu-Rabia and Abu-Rahmoun, 2012; Boudelaa and Marslen-Wilson, 2015; Taha and Saiegh-Haddad, 2016, 2017; Maroun, 2017; Saiegh-Haddad, 2017; Abu Rabia, 2019; Aljasser, 2020; El Akiki and Content, 2020; Hermena and Reichle, 2020; Wattad and Abu-Rabia, 2020; Abu-Rabia, 2021; Alseraye, 2022; Khateb et al., 2022; Aldholmi and Pycha, 2023).

The question became whether adding appropriate short vowels and/or diacritics to the initial HP-HG words would help in blocking the GP phenomenon and enhance the reading process by minimizing reading times and speeding up the parser’s checking processes. In response to the question, Seraye (2004) compared four reading conditions: plain (rc1), short vowels-plus-shaddah (rc2), sukun-only (rc3), and case-ending marking-only (rc4).

The analyses showed no significant results regarding reading conditions for either reading time (*p* = 0.283) or reading comprehension (*p* = 0.237). Examining the total means visually shows that the participants took more time to read rc2 (*M* = 7,277.76) and rc4 (*M* = 7,230.64), and less time to read rc1 (*M* = 6,747.14). For their performance on reading comprehension questions, the percentages of their correct answers were on average very good despite the reading condition (the correct answers percentages range is between 0.89, for rc1, and 0.80, for rc2).

Using the eye movement technique, and with special types of sentences (passive voice), Hermena et al. (2015) examined the effect of Arabic orthographic representation on the reading processes of 25 adult native Arabic speakers, collecting eye movements measures/data on different regions of the sentence. Five reading conditions were constructed by manipulating either the initial HP-HG word or the entire clause, that is embedded in a very complex structural sentence, as exemplified and illustrated above. Only when the initial HP-HG word of the clause was passive and presented as plain would the reader be garden-pathed.

Among the findings revealed by Hermena et al.’s (2015) study, is that Arab adults takes more time to read GP sentences (embedded clause), and that their reading comprehension was not affected (the correct answers percentages range is between 70 and 100%). In addition, a longer fixation duration was observed on the disambiguating region of the GP sentence once the HP-HG initial of the GP sentence was a passive verb and presented as plain.

Since the population in previous studies included highly skilled adult readers, the explanation that relates to experience in the previous findings would garner more support if less experienced Arab readers were incorporated. Therefore, the target sample in Alseraye’s (2022) follow-up study was beginning Arab readers. A total of 39 fourth-grade native Arabic speakers, at the age of 9–10, were included. With the same self-paced moving window software program used by Seraye (2004), the participants read 36 actual seven-word sentences (of both, GP and non-GP sentences) and eight practice sentences representing three reading conditions. These included a plain condition in which only the consonants were presented, a fully vowelized and diacriticized condition, and an incorrectly vowelized condition by manipulating the short vowels only incorrectly while keeping the consonants intact. After reading each sentence, a comprehension question would pop up with three response options: *true*, *false*, and *I do not know*.

The analysis did not reveal any significant differences between the GP and non-GP sentences on reading times (*p* = 0.710) or reading comprehension (*p* = 0.105).

However, examining the overall means showed that it took the participants longer on average to read the GP sentences (*M* = 8,172 ms) than the non-GP ones (*M* = 8,113 ms). For reading comprehension, the overall means for the non-GP and GP sentences were *M* = 0.73 and *M* = 0.80, respectively. Furthermore, when the GP sentences (in the plain condition) were compared to the other two reading conditions, no significant results were found, indicating that the participants’ reading times were the same on average (*p* = 0.565). However, examining the overall mean values showed the following: it took the participants 8,172.33 ms on average to read the GP plain sentences, 8,007.64 ms to read the GP vowelized and diacritized sentences, and 7,882.74 ms to read the GP wrongly vowelized sentences. The overall means showed that it took the participants more time on average to read the plain sentences than the vowelized-diacritized ones. However, the participants benefited from the presence of the short vowels and diacritics that resolved the GP structure; they took less time to read these in comparison to the plain reading condition. Since they took far less time to read the GP sentences that included incorrect short vowels and diacritics, this should have had no effect on their reading time as in the plain reading condition. However, this was not the case. The findings were objectivized as equivocal findings, and the only trend that could be extracted from the results regarding the reading times is that the GP sentences in plain representation took the Arab readers longer to process, regardless of their reading levels (skilled versus beginning). This is consistent with previous findings.

On the other hand, the analysis of the data on reading comprehension revealed a significant difference between the three reading conditions (*p* = 0.026). Pairwise comparisons showed significant differences between reading condition 1 and reading condition 2, (*p* = 0.045) and reading condition 1 and reading condition 3 (*p* = 0.012). However, there was no significant difference between reading condition 2 and reading condition 3 (*p* = 0.618). Examining the means values, however, shows that the participants scored higher on average on condition 1 (*M* = 0.80) than on condition 2 (*M* = 0.69) and condition 3 (*M* = 0.66). This finding that the participants understood the GP plain sentences better than their counterparts in the other reading conditions, is consistent with previous studies (Seraye, 2004; Alseraye, 2022). The trend noted from the previous studies on reading comprehension is that on average the participants understood the GP sentences better than their non-GP counterparts, and that a correlation, from a descriptive perspective, could be inferred visually between the reading times and reading comprehensions; that is, the more time readers of Arabic spend reading the GP, the more accurate responses they score.

In conclusion, the reading behaviors of Arab adults and children regarding the GP sentences showed the following: the persistence of initiating the active basic form of each HP-HG word by making it a default despite its orthographical representation; an automatic attempt to convert the gerund into an active basic verb; and finally, ignoring the supplemental short vowels and diacritics. Furthermore, the statistical results of the previous studies on the GP sentences showed that Arab readers, both adults and children, can read and comprehend the print even if it is presented incompletely, and that they do not need to process the GP sentences twice to comprehend them. Taking the findings of the descriptive and statistical data together leads to questions about the characteristics of the Arabic parser, particularly in terms of the apparatuses that are relied upon in analyzing a consonantal representation of Arabic.

The only factor that can still be suggested as being implicated in the processing of GP sentences in Seraye (2004) and Alseraye (2022) is the reading experience. Therefore, there is likely to be a factor that is involved with and precedes the visual processing of print and that interferes automatically with the visual processing of print even when the writing systems used do not represent speech accurately and completely by vowelizing and diacritizing. It is suggested that this factor is the reader’s previous exposure to print: the reading experience. Indeed, there is evidence to support the belief that people’s previous experiences with linguistic and non-linguistic input play a central role and “strongly shape” their online interpretations of ambiguity in sentences (MacDonald and Hsiao, 2018, p. 176; Alseraye, 2022).

Therefore, we hope that incorporating these L2 Arabic learners as a target population in this continuous research will be helpful in determining the contribution of the two essential explanatory paradigms in sentence parsing/comprehension. This includes the innate explanation (morphological knowledge and word patterns) and the experiential explanation (reading exposure).

The question, then, is, “to what extent the learners of Arabic as a second language, in their advanced competency level, are affected by the incomplete representation of speech (the absence of short vowels and diacritics) in processing GP sentences, their reading time and comprehension.” The response should help to uncover the characteristics of the Arabic parser, by determining to what degree the experience factor is an essential variable by itself or in collaboration with the innate variable that plays a major role in reading ambiguous sentences such as the GP sentences. Therefore, the current study targeted Arabic learners with the justification that their reading experiences with Arabic are evolving, and that this should shed some light on the role of experience in reading Arabic ambiguous sentences.

## Method

### Participants

For the purpose of the study, the sampling technique was judgmental/purposive. A total of 41 participated in the study: 36 of whom were advanced, non-native male Arabic learners of different nationalities, aged from 22 to 26 and enrolled in an Arabic Language Program offered by the Arabic Linguistics Institute at King Saud University, Riyadh, Saudi Arabia. The remaining five participants had already graduated from the Arabic institute.

Initially, to identify participants for the study, three teachers were consulted to assess their students’ language competency on a 5 point rating scale (5-excellent, 4-very good, 3-good, 2-fair, 1-poor). For a sample size consideration, only the participants who were in the 1-poor level were excluded right from the start. According to the rating value means, the participants ranged in language competency between 3 and 5, with only 4 out of the 34 participants were rated less than 4. The overall mean was 4.38, with a standard deviation equals 0.652.

The participants were all offered R80 ($20) as compensation for participating. The data that were later used for the analysis included only those participants who demonstrated a reading fluency skill, based on the post critierain assessment (reading aloud task) that was held after completing the computer task, of whom there were 34. Official approval and consent for participation were obtained beforehand.

### Materials

The same two sets of sentences that were constructed as the stimuli for a previous study (Alseraye, 2022) were used for this study and for a subsequent comparison to native Arabic speakers, to assess assumptions that had been raised by the previous study. The first set of reading conditions included 31 sentences, seven of which were for the practice session, 12 to represent the plain reading condition, rc1, and 12 for the fully vowelized and diacriticized reading condition, rc2. There were four GP sentences in each reading condition. In rc2, when presented as fully vowelized and diacriticized, the short vowels and diacritics on the initial HP-HG words in the sentences would resolve the GP structure only if the readers assembled them with the consonants.

The second set of conditions contained 12 sentences: one for the practice session; the others representing the wrongly vowelized reading condition, rc3. The consonants were supplemented with incorrect short vowels. Four potential GP sentences were included too in this reading condition. For each sentence a textually based comprehension question was constructed that entailed three responses: *true*, *false*, and *I do not know*. The third option, *I do not know*, was given to help the participants avoid having to guess. All the questions were presented fully vowelized and diacriticized.

Only the GP sentences in each condition were the targets, and the remaining stimuli were used as filler items and for a comparison reason.

There were seven words in each sentence. All words were of high frequency, and represent the basic structure Arabic takes, and that Arabic readers encounter in connected texts. The sentences among the three conditions were matched syntactically (see Appendix B). In the GP sentences, approximately three words separated the initial HP-HG word from the disambiguating region (for an example, see Diagram 1). The sentences and questions were already assessed and judged by a team of Arabic fourth-grade teachers and graduate students in teaching Arabic program, and then reassessed for the current study by some graduate students in the program of teaching Arabic as a second language. Assessing the sentences and questions was in terms of naturalness, accuracy, suitability, word familiarity, capability of capturing comprehension, and so on. No change in the original sentences was made (see Appendix A for the sentences and questions used in the experiment).

For a post critierian assessment of reading fluency, and for manifesting what is going on in the L2 Arabic learners’ minds as they approach the GP and the potential/resolved GP sentences (by providing them with the right short vowels and diacritics), an informational/expository text of 170 words of high frequency was constructed for the reading aloud task (see Appendix C). Three GP sentences were inserted in the text. In addition, it included some passive and active HP-HG initials. Two equivalent versions of the same text was constructed. Both versions were the same and presented as plain, except in one version, the HP-HG initials of GP sentences (and the passive sentences) were provided with the right short vowels/diacritics to turn them into a non-GP sentences.

### Measures

The following two dependent variables were measured: reading time, measured to the nearest millisecond, and comprehension product, percentage of correct responses. These were measured and coded as *true*, *false*, and *I do not know*. Each correct answer was assigned a 1; all others, false or I do not know, were given a 0.

### Procedure

The study followed and adopted the same procedure and paced-reading software that was used in Alseraye (2022) study on Arab children.

The procedure took the following format: the participants logged in, viewed the instructions, and then started the reading task, using a button (space-bar key) that showed every word sequentially when they clicked on it but hid the previous ones. Once they had finished and pressed the space-bar key, a question would pop up with the three options for responding. The same process continued through to the final sentence. When the participants did not know the answer, or felt tempted to guess, they were told to choose “*I do not know*.”

They were informed that they would read sentences in which the words were presented with the wrong short vowels, and that assembling the wrong short vowels would lead to constructing words that had no meaning in Arabic; that is, the graphemic form (consonants) of the words was intact, but the phonological aspect was distorted. Assembling only the consonants and ignoring the short vowel signs would result in participants reading a real word in Arabic.

Once the computer-based task was completed, a short reading task was held immediately: a running record by‑ the researcher was applied while the participants were asked to read aloud a short text that included two GP sentences, similar to the ones conducted in the computer task (and passive sentences). The aim of the task was for the participants to manifest their reading behavior once they encounter an ambiguous GP structure, Further, the task serves to determine whether L2 Arabic learners were conscious of the ambiguity of the GP sentences. The reading aloud texts were randomly assigned to the participants: some participants read the completely plain version while the others read the one with the resolved GP sentences.

It is worth mentioning here that, the task was used to further ensure that the selected participants were indeed at an advanced proficiency level.

### Design and analysis

An empirical study with a one-factor within-subjects design was employed to evaluate the effects of the GP structure on it own and in conjunction with short vowels and diacritics on the reading processes of learners of Arabic. Four analyses were conducted, and two separate statistical procedures were employed, the dependent samples *t*-test and the one-way repeated measures analysis of variance. The two tests’ assumptions were checked prior to the analyses (i.e., the level of measurement, normality, homogeneity, outliers, sphericity).

## Results

### Descriptive part

From a descriptive perspective, observing the reading behavior of the Arabic learners revealed very similar patterns that were observed in previous studies conducted on Arabic adults (Seraye, 2004) and children (Alseraye, 2022). However, there was one observation that was unique to the L2 Arabic learners. They would apply what they had already experienced; that is, their background knowledge of the verb form they had just bypassed, to the next verb form they encountered but would subsequently figure out that the verb was in a passive form. This experience was then applied to the next passive verb, which they would get right.

### Statistical part

#### The plain GP/non-GP reading condition subdata

To respond to the concerns raised by a previous study (Alseraye, 2022), three types of analyses were conducted on three subsets of data. In the first analysis, GP and non-GP sentences in the plain representation were compared using a dependent samples *t*-test, to detect whether those who were less experienced with print were affected by the GP structure of the sentences. This involved comparing GP and non-GP sentences in terms of reading time and percentage of correct responses.

##### Reading times analysis

Regarding the data for reading times, the analysis (Table 1) revealed that the GP structure had a significant effect [*t*(34) = −2.15, *p* = 0.039]. The difference in the mean values was roughly 327 ms, which means that it took the participants longer to read the GP sentences than the non-GP sentences (*M* = 6,636 ms for the GP sentences; *M* = 6,309 ms for the non-GP sentences).

**Table 1:** Results of the *t*-test on the reading times of GP and non-GP sentences.

Non-GP sentences		GP sentences		*t*	*df*	*p*
*M*	*SD*	*M*	*SD*			
6,308.6	1,850.6	6,635.6	1,952.1	−2.149	33	0.039

GP, garden path.

Since the analysis included the reading times of both, the correct and incorrect answers data, a subset data of only the correct responses was considered by excluding the incorrect answers from the analysis, in order to have a robust results, using the dependent samples *t*-test. The analysis revealed the same results; a significant effect of the GP sentences (6787.688 ms for the GP sentences vs. 6206.397 ms for the non-GP sentences) on the reading times of the participants [*t*(33) = −2.63, *p* = 0.013]. Due to the existence of outliers, a non-parmetric test was conducted, and the same results were revealed (z = −2.881, *p* = 0.004).

This result is consistent with the findings of previous studies on Arabic native speakers, both adults (Seraye, 2004) and children (Alseraye, 2022), which demonstrated the effect of the garden-path structure on the reading processes for Arabic texts (see Table 2):

**Table 2:** Results of the *t*-tests on the reading times of GP and non-GP sentences between the three populations.

Population		Non-GP sentences		GP sentences		*p*
		*M*	*SD*	*M*	*SD*	
*L2 Arabic*	*Learners*	6,308.6	1,850.6	6,635.6	1,952.1	0.039
*Arab*	*Children*	8,112.7	2,380.6	8,172.3	2,486.3	0.710
*Arab*	*Adults*	6,259.3	1,413.3	6,747.1	2,071.9	0.016

Based on the overall means for the L2 Arabic learners, Arab children, and Arab adults, we found that the GP structure influenced the reading processes of Arabic readers, regardless of their reading levels, reading experience, and print exposure. Another observation concerned the total time spent reading the GP sentences; the Arab adults and the L2 Arabic learners took roughly the same amounts of time to read these sentences (i.e., the same trend). Note, however, that they read different sets of sentences in terms of length and the distance between the HP-HG initial of the GP sentences and their ambiguating region. According to the literature, the decrease in distance between the initial word of the sentence and its disambiguating region should positively affect the reanalysis of the GP sentences (Ferreira and Henderson, 1998; Ferreira et al., 2001), and help to keep the essential parts of the sentence active.

##### Reading comprehension analysis

In terms of the data for the reading comprehension, the analysis did not reveal any significant differences between the means for the percentages of correct responses for either type of sentences [*t*(33) = −0.362, *p* = 0.720] (see Table 3). The correct responses of the participants did not, on average, differ significantly between the GP and non-GP sentences (the overall mean for the non-GP sentences was *M* = 0.86; the overall mean for the GP sentences was *M* = 0.88).

**Table 3:** Results of the *t*-test on the reading comprehension of GP and non-GP sentences.

Non-GP sentences		GP sentences		*t*	*df*	*p*
*M*	*SD*	*M*	*SD*			
0.86	0.17	0.88	0.23	−0.362	33	0.720

GP, garden path.

However, because the data on comprehension were extremely skewed, since the participants’ comprehension was generally very good and because of the outliers, a non-parametric test, the Wilcoxon matched-pair signed-rank test, was used along with the dependent samples *t*-test analysis. However, the analysis did not reveal any significant differences between the two means, *z* values (−1.023), and *p-*values (0.306); therefore, only the result of the *t*-test is provided in Table 4.

**Table 4:** Results of the *t*-tests on the reading comprehension of GP and non-GP sentences between the three populations.

Population		Non-GP sentences		GP sentences		*p*
		*M*	*SD*	*M*	*SD*	
*L2 Arabic*	*Learners*	0.86	0.17	0.88	0.23	0.720
*Arab*	*Children*	0.73	0.22	0.80	0.32	0.105
*Arab*	*Adults*	0.83	0.08	0.89	0.17	0.053

These results, which show no significant differences between the two types of sentences, are consistent with those of Seraye’s two studies of highly skilled adult readers and children who are beginning readers (2004) and (Alseraye, 2022) respectively). Although the GP structure affected reading processing by adding more time loads onto the process, the readers’ comprehension was not affected: they had higher scores regardless of the type of structure they processed (Table 4).

The same pattern that emerged from the data for the reading times on the three populations is observed with the comprehension data. The adults, both Arabs and non-Arabs, had higher scores on average on the GP sentences than the Arab children. In addition, the difference between the two means was less among L2 Arabic learners, with only a 2% difference. However, among the Arab adults and Arab children the difference was nearly 7%.

Descriptively, when aligning the comprehension data with the reading time data, a pattern emerges that suggests a relationship between the two (Tables 3, 4).

#### The GP three reading conditions data

The comprehension issues with the GP sentences were addressed by adding the correct short vowels and diacritics to the initial HP-HG words. To further explore this, a one-way repeated measures analysis of variance was conducted specifically on a subset of the data. This aimed to answer whether the inclusion of short vowels and diacritics would significantly affect the reading process of L2 Arabic learners.

For the GP and potential GP sentences, we compared reading times and comprehension across three different reading conditions, including a control condition with incorrect vowelization. The control was included to determine if the addition of incorrect short vowels would impact reading, suggesting that L2 Arabic learners may not rely on sub- and superscript processing, but rather on consonant processing (Seraye, 2004). The central question was whether the addition of short vowels and diacritics would be beneficial in the reading process of GP sentences by L2 Arabic learners.

##### Reading times analysis

In terms of the data for the reading times, the assumption of sphericity (using Mauchly’s Test of Sphericity) was examined first and found to be significant. Therefore, the condition of sphericity was not met, and a nonparametric test was used in addition to the parametric one.

**Figure 1:**
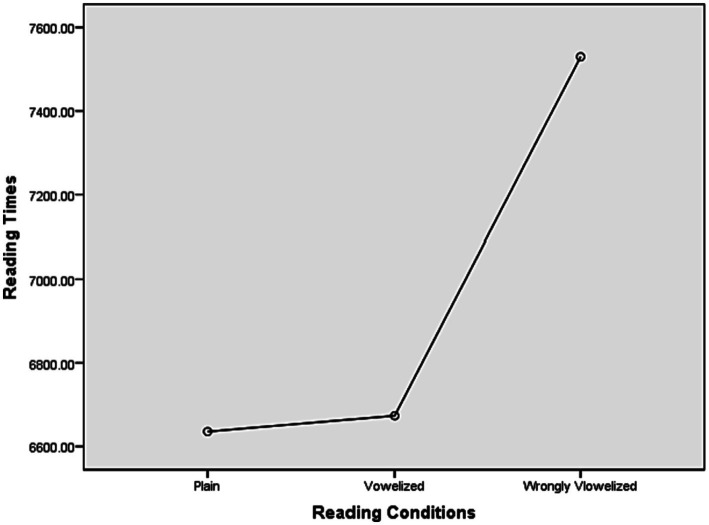
Overall means on reading time for GP sentences on three reading conditions.

By first using the parametric test, the analysis on the data for reading times revealed a significant difference between the reading conditions (*F* (2, 60) = 8.293, *p* = 0.003). It took the participants on average 6,514.55 ms to read the GP sentences that were presented as plain ones, 6,640.84 ms to read the GP sentences that were supplemented with the correct short vowels and diacritics, and 7,446.72 ms to read the GP sentences that were supplemented with incorrect short vowels (Table 5 and Figure 1).

**Table 5:** Overall means on reading time for GP sentences.

	Reading condition (sentence stimuli)	GP sentences	
		*M*	*SD*
Group	Plain (no short vowels or diacritics)	6514.55	1986.81
	Fully vowelized and diacritized	6640.84	1887.05
	Wrong short vowels	7446.72	2430.67

Furthermore, a non-parametric test (Friedman Test) showed a significant difference between the mean ranks, ꭓ(2) (Chi-Square) = 6.258, *p* = 0.044.

However, the overall means showed that it took the participants less time on average to read the plain sentences than their vowelized-diacritized counterparts (126 ms difference). These means also showed that participants took more time on average to read the vowelized condition that was incorrect (see Table 5).

For the same reason mentioned above, the incorrect answer data were excluded from the analysis, using a one-way repeated measures analysis of variance. The analysis of the reading times for the GP sentences revealed the same results; a significant effect of the GP sentences between the reading conditions [*F*(2, 60) = 3.749, *p* = 0.044]. Due to the violation of the test assumption (Mauchly’s Test of Sphericity), a nonparametric test (Friedman Test) was conducted, revealing nonsignificant difference between the mean ranks, ꭓ(2) (Chi-Square) = 5.871, *p* = 053. Examining the total means visually shows, however, that the participants took on average less time to read rc1 (*M* = 6,635.97; SD = 1980.18) than rc2 (*M* = 6,687.60; SD = 2075.70), and much less than rc3 (*M* = 7,429.26; SD = 2529.75). Indeed, although it was not specifically an aim in the current study, examining the raw data of only the disambiguating region, word-6 by itself, in the three GP reading conditions and the non-GP plain reading condition showed that the participants, on average, spent longer reading (fixating) the word-6 (*M* = 948.60 ms) relative to its counterparts in the non-GP plain sentences (880.09 ms) and the two reading conditions (*M* = 892.25 m, in rc2; *M* = 849.12, in rc3).

This result is not consistent with Alseraye’s (2022) study of Arab children, which showed that they took on average more time to read the plain reading condition and less time to read the vowelized counterparts that were incorrect (Table 6). The data suggest, then, that L2 Arabic learners do not benefit from the short vowels in processing the ambiguous sentences (i.e., GP), but that they were influenced by the effects that the wrong short vowels had on the consonants. As they reported after their sessions reading aloud, this interfered with their reading processes, and they found it difficult to ignore the short vowels and diacritics. This interference was observed among both the native and the non-native participants. As the mean values in Table 6 show, the adults took more time to read the GP sentences that were resolved by the right diacritics.

**Table 6:** Results of the repeated measures analysis of variance on the reading times of GP sentences between the three populations.

Population		Plain condition	Vowelized condition	Wrongly vowelized *p*-value condition	
		*M*	*M*	*M*	
*Arabic*	*Learners (current study)*	6514.6	6640.8	7446.7	*p* = 0.044
*Arab*	*Children* (Alseraye, 2022)	8172.3	8007.6	7882.7	*p* = 0.565
*Arab*	*Adults* (Seraye, 2004)	6747.1	6997.3		
			7230.6*		

*These reading time values were for reading conditions in which the GP sentences were provided with only the right diacritics, either a sukun or a case ending (i.e., not vowelized completely) that when assembled would resolve the ambiguity by turning the GP sentence into a non-GP counterpart.

##### Reading comprehension analysis

**Figure 2:**
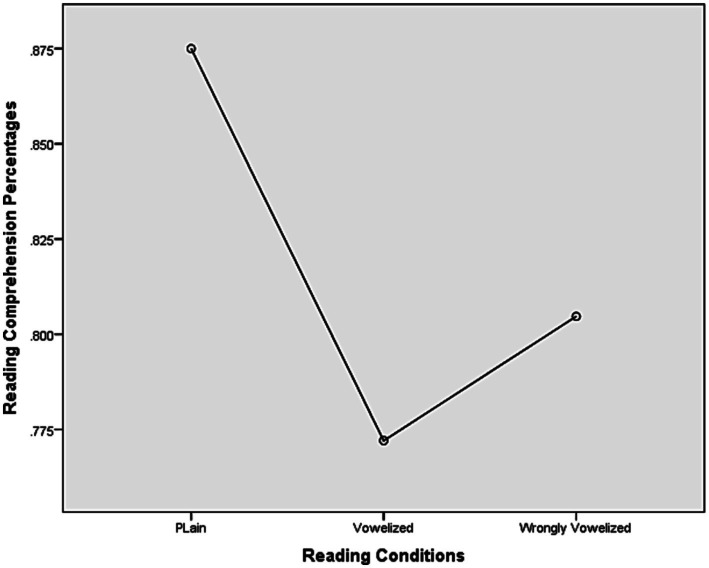
Overall means on reading comprehension for GP sentences on three reading conditions.

The assumption of sphericity was examined for the reading comprehension data and found not to be significant; the condition of sphericity was met. The repeated measures analysis of variance revealed no significant difference between the three reading conditions [*F*(2, 60) = 3.109, *p* = 0.052]. However, by accepting that the *p* value was nearly significant, the pairwise comparisons showed that the only significant difference was between rc1, the plain one, and rc2, the vowelized one (*p* = 0.017). On average, the participants scored best on the plain reading condition (*M* = 0.89), and worst on the vowelized one (*M* = 0.77) (Table 7 and Figure 2).

**Table 7:** Overall means on reading comprehension for GP sentences.

	Reading condition (sentence stimuli)	GP sentences	
		*M*	*SD*
Group	Plain (no short vowels or diacritics)	0.89	0.21
	Short vowels-plus-diacritics	0.77	0.24
	Wrong short vowels	0.80	0.21

Since the data were not normally distributed, a non-parametric test was run (Friedman Test), revealing a significant difference between the mean ranks, ꭓ(2) (Chi-Square) = 6.077, *p* = 0.048.

This result, as shown in the raw means in Tables 7, 8, is not completely consistent with the previous studies. The only consistency observed is that the readers of Arabic, native and non-native alike, scored higher when the GP sentences were presented only in consonant form (plain rc1), and that adding the resolving short vowels and diacritics to the consonants of the GP sentences showed no benefits.

**Table 8:** Results of the repeated measures analysis of variance on the reading comprehension of GP among the three populations.

Population		Plain condition	Vowelized condition	Wrongly vowelized *p*-value condition	
		*M*	*M*	*M*	
*L2 Arabic*	*Learners (current study)*	0.86	0.73	0.83	*p* = 0.048
*Arab*	*Children* (Seraye, 2022)	0.80	0.69	0.66	*p* = 0.026
*Arab*	*Adults* (Seraye, 2004)	0.89	0.88*.	.	
			86		

*These reading comprehension values in the Arabic adult study were for a reading condition in which the GP sentences were provided with only the right diacritics, either a sukun or a case ending (i.e., not vowelized completely), that when assembled would resolve the ambiguity by turning the GP sentence into a non-GP counterpart.

## Discussion

Essentially, there are two general observations that summarize the data for reading times and reading comprehension in a consistent manner (Tables 4, 6, 8). The first is that the readers of Arabic spent less time on average reading GP sentences in plain reading conditions, where only the consonants were presented, and that providing them with short vowels and diacritics seems to introduce a disturbing factor by increasing their reading times. This result was reached through visual examination by Seraye’s (2004) study of highly skilled Arab adults that revealed the following: “the more the short vowels and *shaddah* signs were provided, the more time it took the participants to read the sentences” (p. 181). This effect that the GP structure had on the reading process, as reflected in the extra time required to read this type of structure, is well documented across different orthographies and various writing systems. It can be explained according to two assumptions: “on the basis of the implicit checking process that operates with a delay cost or on the basis of the processing load in the ambiguous region.” The effects were demonstrated by several other studies that used different techniques such as eye-tracking studies (Roman et al., 1985; Ferreira and Henderson, 1990, Experiment 1; Hermena et al., 2015), first fixation data (Frazier and Rayner, 1982), self-paced reading tasks (Mitchell et al., 1992, Experiment 1, as cited in Mitchell, 1994, p. 381; Seraye, 2004, Experiment 1; Alseraye, 2022), and brain imaging (Mason et al., 2003).

The second general observation is that the readers of Arabic scored higher on average on the plain reading condition than for the other conditions (Tables 6, 8). Although providing the consonants with the right short vowels and diacritics should at least block the GP phenomenon and therefore decrease the reading time of the GP sentences by reducing the hesitancy/reluctance over the disambiguating region, this was not the case. The question then arose regarding the mechanism that helped both the novice and experienced readers to understand GP sentences that were run in a self-paced reading software program design that prevented them from returning to earlier parts of the sentences to clarify or verify their understanding of the GP sentences. One recurrent explanation attributes the good performance to the fact that the readers rely on the richness of Arabic morphology and the pattern, form, and roots in which the words are constituted on either three- or four-root skeletons.

Arabic morphology, which is centered around a trilateral/quadrilateral root system, suggests that Arab readers, when presented with a consonant-based script, are expected to utilize their knowledge of Arabic word formation in accessing mental lexicon representations (Abu-Rabia, 1995–2001). Seraye (2004) elaborates that within the array of activated potential word forms, there is often a shared trilateral/quadrilateral root indicating a central semantic element, while the word form or pattern (its skeletal tier/word pattern/binyan, McCarthy, 1979, 1981) restricts the potential readings of the word. This structural predictability and the productivity of word forms and affixation are what compensate for the lack of short vowels and diacritics in the written language.

The role of Arabic morphology roots in the reading process has recently, as noted earlier, been revisited, consolidated, and thoroughly documented (Alseraye, 2022, p. 17). Indeed, the investigation was recently directed toward concerns about the Arabic lexicon representation, the classes of morphological representation (roots vs. words patterns: nominal and verbal), and the degree to which the process of naming words could be facilitated (see, for example, Aljasser, 2020; Khateb et al., 2022; Aldholmi and Pycha, 2023).

However, the insignificant role of short vowels and diacritics in the reading processing of GP sentences can also be explained according to two assumptions:

“by the fact that subjects, as Ferreira et al. (2009) state, ‘have a tendency to sacrifice reanalysis of the garden-path in order to keep up with later material. This pattern of results is consistent with the assumptions of the good enough theory of language processing, which assumes that processing resources are limited, and therefore predicts that garden-path reanalysis processes will be curtailed if upcoming material must also be processed” (p. 416).

As noted earlier, giving up the reanalysis of the GP structure was observed visually during the task involving reading aloud. The participants, Arab adults, Arab children, and L2 Arabic learners, did not go back to reanalyze the GP sentences even when they knew that their initial interpretations of the GP sentences were wrong, although some responded to their mistakes by making exclamations such as, “لا!” meaning “no!” However, the claim that there is no reanalysis was observed was based on the fact that the participants, Arabs and none Arabs, both children and adults, never went back to reread the HP-HG verb, and choose its right form. Indeed, the reanalysis could have been occurred with no trace of verbalizing it, which cannot be examined by using a reading aloud task. Only with an eye-movement technique, such a claim can be assuredly assessed.

The second assumption involves previous reading experience, which is further implicated in the equivocal results (Alseraye, 2022). Monitoring the reading behavior of the Arab and non-Arab readers of the language during the task involving reading aloud demonstrates that they apply what they have already experienced to the next verb form they meet in the text.

Another explanation that can be presented here, and which is supported by the data of both the current study and previous studies by Seraye (2004) and Alseraye (2022) is related to predictions. That is, because of the segregability representation of Arabic writing system, and the absence of short vowels and diacritics, the readers would expect to be able to predict what is next, based on the semantics and syntax of the language during reading an HP-HG initial sentence. In the Arabic case, the readers are expected to use their knowledge of the semantic and syntactic features in predicting what follows. Within the sentence parsing models (Left-Corner parsing, the Garden-Path model, Syntactic Prediction Locality Theory (SPLT), Good-Enough and Noisy Channel processing, and Surprisal and Entropy in Information-Theoretic models of language processing), prediction is considered a central component in modeling human sentence parsing (Ferreira and Qiu, 2021). Although both semantics and syntax are clear predictors in explaining the situation, the syntactic prediction in the case of Arabic seems to be strong logically and through observation because of the different characteristics of the language, including its morphological features. These include the dominant word order, the features of the writing style (anastrophe), and the inversion of the word orders in phrases and clauses resulting from the disappearance of grammatical case endings, which would force the writer to avoid any disturbance that could arise through this disappearance. Furthermore, in any modern Arabic writing, the distance between the subject and its predicate is not too far to put a load on the reader’s memory. To illustrate, the transitive forms of the verbs are sometimes used as intransitive forms, and prepositions are attached to the NPs to help the readers to grasp the focus in advance and avoid any associated disturbance. Arabic writing includes many transitive verbs that are currently used as intransitive ones. For example, there is the verb “قَبِلَ” meaning “[He] accepted …,” in which the transitive verb has become intransitive in journalistic writing [for more details on this issue, see Afifi Ahmed’s (2004) study].

Also, with the passive voice for verbs, although Arabic allows both aspects, the active and passive voices, it is expected that the stylistic features of the discourse help in finding the aspect voice of the verb. Indeed, even having the verb next to its subject would be close enough to prevent the ambiguity, particularly with respect to the GP phenomenon. However, using the passive voice, where the GP phenomenon would be obvious, is not really encouraged unless the context requires it. Furthermore, in modern Arabic writing, alien/outlandish expressions can leak into the writers’ linguistic expressions when they want to avoid using the passive voice by inserting the word “تَمَّ” which means “was completed,” which acts as a suffix, and converting the main verb “كسر” into a gerund. This, then, means that the object “الكرسي the chair” was acted upon by some other performer of the verb, or that they may use other morphological devices by changing the patterns of the verbs, while keeping the meaning unchanged, such as through the form pattern “انكسر broke itself” or using the topicalization mechanism (see the examples below):

_____________________________________________________

خالد كسر الكرسي Khalid broke the chair.

الكرسي كُسر بواسطة خالد/ كُسِر الكرسي بواسطة خالد The chair was broken by Khalid.

تم كسر الكرسي The chair has been broken.

انكسر الكرسي The chair was broken.

_____________________________________________________

Clearly, this type of writing representation would affect readers, and they would, therefore, expect to follow a trend of reading behavior. The extended exposure to the current print as it is usually represented would be likely to build a determined experience in its readers that would eventually help them to construct in their minds a faculty of prediction, which would then show in their reading behaviors. This appears reasonable because of the incompleteness of speech in Arabic print that is due to the absence of short vowels and diacritics from the print. Using this type of experience would help prepare the reader to emphasize some sensory inputs and ignore others. That is, the cognition of the experienced readers of Arabic, to apply Gibson’s theoretical framework of perception, would be built on a foundation of perceptual knowledge that would become a system of representation about the verbal sentences that begins with an HP-HG initial word (Gibson, 1988). Indeed, the constructivist view of perception presented by Gregory (1997) might be a good grounding explanation for what the Arab readers do while processing HP-HG initial sentences. That is, a top-down perceptive is emphasized in which the visual perception of Arabic readers uses inferences from visual cues and past experience during processing HP-HG initial sentences.

To illustrate this, we observed that both native and L2 Arabic learners read the initial HP-HG verbal word of a sentence as a verb in the active voice (the default in their minds), even when the initial verbal word was supplied with the necessary short vowels and diacritics. Therefore, because speech is less represented in Arabic orthography, the parser would be trained to rely on past experiences (e.g., frequent exposures) and approach those homographic-initial word sentences first.

Based on the findings of this study, it is recommended that different techniques to be used on the same sentence stimuli, to consolidate or refute the current findings as, for example, by adopting the eye movement technique. Other recommendations are the adoption of a qualitative approach in figuring out what is going on in the Arabic readers’ mind, as they approach and resolve the ambiguity caused by the GP sentences, in their two types of representations: vowelized and non-vowelized text representations. That is, by using the “Think Aloud” procedure, the participants should be asked to verbalize their thinking processes as they read GP sentences. It is also recommended that different populations to be targeted; for example, by investigating the effect of the GP phenomenon on less skilled readers and on participants who have reading difficulties, such as dyslexia.

Pedagogically, the ambiguity resulting from the omission of short vowels and diacritics should be addressed in Arabic teaching contexts. Given their ubiquitous absence in printed material, it is beneficial for Arabic learners to practice reading texts, particularly the HP-HG initial sentences, with such orthographic characteristics. Furthermore, students should be taught to apply short vowels and diacritics both economically and efficiently in their own writing.

## Data availability statement

The raw data supporting the conclusions of this article will be made available by the authors, without undue reservation.

## Ethics statement

The studies involving humans were approved by Research Ethics Committee-Vice Rectorate for Graduate Studies & Scientific Research-Deanship of Scientific Research-King Saud University. The studies were conducted in accordance with the local legislation and institutional requirements. The participants provided their written informed consent to participate in this study.

## Author contributions

AA: Data curation, Formal analysis, Methodology, Supervision, Writing – original draft, Investigation, Project administration, Writing – review & editing.
