# Corrigendum: L2 Arabic learners' processing of Arabic garden-path sentences: a consistent reading pattern

**DOI:** 10.3389/fpsyg.2024.1414363

**Published:** 2024-04-22

**Authors:** Abdullah M. Seraye Alseraye

**Affiliations:** Department of Curriculum & Instruction, School of Education, King Saud University, Riyadh, Saudi Arabia

**Keywords:** garden-path structure, Arabic short vowels, past experience, heterophonic-homographic initial, L2 Arabic learners, reading comprehension

In the published article, there was an error. The author realized that a mistake was made during the process of writing the article. The author name quoted, “Ots,” was repeated in the next sentence, leading to a claim being mistakenly attributed to this author. A correction has been made to clarify the text and amend the mistaken attribution.

A correction has been made to *Introduction, Paragraph 6*. The corrected sentence appears below:

According to Ots (2021), the speaker, in the linguistic encoding stage in language production, would “assign the syntactic functions [that] are appropriate for the message and order the constituents, given the discourse and grammatical constraints” (p. 2). Similarly, the writer, I assume, would be forced to comply with these constraints, in addition to the cognitive constraints and limitations, to avoid the long distance between the HP-HG word and its disambiguating region in the sentence.

The author apologizes for this error and states that this does not change the scientific conclusions of the article in any way. The original article has been updated.

