# Supplementary material for: L2 Arabic learners’ processing of Arabic garden-path sentences: a consistent reading pattern
Source: Front Psychol. 2024 Mar 8;15:1333112. doi: 10.3389/fpsyg.2024.1333112 (PMC10957760; doi:10.3389/fpsyg.2024.1333112)
Supplement: Supplementary file 1 [file Table_1.pdf]

Appendix A

The experimental items: Sentences and questions
Set I (RC1: Plain; and RC2: Vowels + diacritics, sentences)

Questions/answers	Plain; and Vowels + diacritics, sentences
	Practice sentences (1 – 7)
هَلْ يُحِبُّ مُحّمَّدٌ أَنْ يَلْعَبَ بِالْكُرَةِ كُلَّ يَوْمٍ ؟<br>1	1. مُحّمَّدٌ يُحِبُّ أَنْ يَلْعَبَ بِالْكُرَةِ كُلَّ يَوْمٍ.
خَالِدٌ يُحِبُّ أَكْلَ التُّفَّاحَ؟<br>0	2. خَالِدٌ لاَ يُحِبُّ أَكْلَ التُّفَّاحَ أَبَداً.
رَاشِدٌ اِبْتَعَدَ عَنْ بَلَدِهِ لِمُدَّةٍ قَصِيْرَةٍ؟<br>0	3. جَاءَ رَاشِدٌ مِنْ سَفَرِهِ بَعْدَ غِيابٍ طَوِيْلٍ.
الإِكْثَارُ مِنْ عَصِيْرِ الْلَيْمُونِ يَضّرُّ بِالأَسْنَانِ؟<br>0	4. الإِكْثَارُ مِنْ عَصِيْرِ الْلَيْمُونِ لاَ يَضرُّ بِالأَسْنَان.
شَاهَدَ عَبْدُاللَّهِ بَرْنامَجاً عَنْ مَخَاطِرِ الحُرُوْبِ ؟<br>0	5. شَاهَدَ عَبْدُاللَّهِ بَرْنَامَجَاً تِلْفَازِيَّاً عَنْ أَضْرارِ التَّدْخِينِ.
هَلْ حَصَلَ مُنْتَخَبُ كُرَةِ الطَّائِرَة عَلَى المَرْكَزِ الثَّانِي؟<br>0	6. حَصَلَ مُنْتَخَبُ كُرَةِ الطَّائِرَة عَلَى المَرْكَزِ الثَّالِثِ.
هَلْ احترقت كتب خالد بسبب الحريق؟<br>0	7. كتب خالد الموجودة في المكتبة لم تحترق.
	Plain sentences (8 – 19)
الَّذِي رَسَمَ الصُّورَةَ الْجَمِيْلَةَ كَانَ تِلْمِيْذاً صَغِيْرَاً؟<br>0	8. الذي رسم الصورة الجميلة كان رجلا عجوزا.
الْبَيْعُ وَالشِّرَاءُ بِجَانِبِ مَبْنَى الْوَزَارَةِ مَمْنُوعٌ؟<br>0	9. إن البيع والشراء بجانب مبنى الوزارة مسموح.
اِسْتِقْدَامُ الْعَامِلاَتِ مِنْ أَي بَلَدٍ أَمْرٌ يَسِيرٌ؟<br>1	10. استقدام العاملات من كل البلدان أصبح يسيرا.
أَتَى خَالِدٌ مِن السُّوقِ رَاكِبَاً سَيَّارَةً؟<br>0	11. جاء خالد من السوق ماشيا على قدميه.
السَّهَرُ كَانَ مِنْ أَسْبَابِ مَرَضِ مُحَمَّدٍ؟<br>1	12. من أسباب مرض محمد عدم نومه ليلا.
هَلْ ذَاكَرَ الاِبْنُ دُرُوسَهُ ؟<br>0	13. غضب الأب من ابنه لأنه لم يذاكر.
خَوْفُ السُّكَّانِ كَانَ بِسَبَبِ صَوَاعِقَ شَدِيْدَةٍ؟<br>0	14. حدث خوف للسكان بعد حصول الزلزال القوي.
سَعْدٌ هُوَ الْمُعَلِّمُ لِخَالِد؟<br>0	15. علم خالد أخاه سعدا كتابة حروف العربية.
	Garden-path sentences (16 - 19)
البَائِعُ فَتَحَ مَحِلَّهُ فِيْ وَقْتِ الصَّبَاحِ وَلَكِن مُتَأَخِّراً؟<br>0	16. فتح البائع محله في الصباح كان مبكرا GP.
أَحْمَدُ رَجُلٌ سَلِيْمٌ لاَ يَشْتَكِي مِنْ أَيِّ أَمْرَاضٍ؟<br>0	17. مرض أحمد بمرض السكري كان لكثرة جلوسه GP.
أَزْعَجَ الأَطْفَالُ السُّكَّانَ بِلَعِبِهِم الْكُرَة صَبَاحاً؟<br>0	18. لعب الأطفال بالكرة صباحا لم يزعج السكان GP.
الطَّبِيْبُ كَشَفَ عَلَى خَالِدٍ مِنْ غَيْرِ مُسَاعَدَةِ أَحَدٍ؟<br>0	19. كشف محمد مرض خالد كان بمساعدة الممرضة GP.
	Vowelized-diacritized sentences (20 – 31)
	20. الذَّي كَتَبَ الْقِصَّة الْجَمِيْلَة كَان طِفْلَا صَغِيْرا.
	21. إِنَّ اللَّعِب وَالسِّبَاق بِجَانِب مَبْنَى الْوَزَارَة مَسْمُوح.
	22. اِخْتِبَار الطُّلاَّب فِي مَادَّة الْقِرَاءَة كَان صَعْبا.
	23. جَاء صَالِح مِن الْعَمَل مُسْرِعا فِي سَيَّارَته.
	24. مِن أَسْبَاب مَرَض خَالِد عَدَم أَكْله الْخُضْرَوَات.
	25. صَرَخ الأَب عَلَى اِبْنه لأَنَّه لَم يَسْتَمِع.
	26. فَرّ رِجَال الإِطْفَاء بَعْد سُقُوط الْجِدَار الطَّوِيْل.
	27. دَرَّس فَهْد صَدِيْقه أَحْمَد كِتَابَة اِسْمِه بِالإِنْجِلِيْزِيَّة.
	Potentially Garden-path sentences (28 – 31)

هَلْ الَّذِي كَتَبَ الْقِصَّةَ كَانَ طِفْلا صَغِيْرَاً؟<br>1<br>اللِّعْبُ وَالسِّبَّاقُ بِجَانِبِ مَبْنَى الْوَزَرَاةِ مَمْنُوعٌ؟<br>0<br>الاِخْتِبَارُ فِي مَادَّةِ الْقِرَاءَةِ كَانَ سَهْلَا؟<br>0<br>قَادَ صَالِحٌ السَّيَّارَةَ بِسُرْعَةٍ؟<br>1<br>خَالِدٌ لاَ يُحِبُّ أَكْلَ الْخُضْرَوَاتِ؟<br>1<br>اِبْتَسَمَ الأَبُ لابْنِهِ لأَنَّهُ كَانَ يَسْتَمْتِعَ بِلُعْبَتِهِ؟<br>0<br>هُرُوبُ رِجَالِ الإِطْفَاءِ كَانَ قَبْلَ سُقُوطِ الجِدَارُ؟<br>0<br>فَهْدٌ يَعْرِفُ الإِنْجِلِيْزِيَّةَ أَكْثَرَ مِنْ أَحْمَدَ؟<br>0<br>خَالِدٌ يَعْرِفُ كَيْفَ يَرْبُطُ أَسْلاَكَ الْكَهْرَبَاءِ؟<br>0<br>لَمْ يَجِدْ أَحْمَدُ مَفَاتِيْحَ الْمَحَلِ, فَقَامَ بِكَسْرِ الْقُفْلِ؟<br>1<br>لاَ يَسْتَطِيْعُ أَحْمَدُ تَنَاولَ عَصِيْرِ الْبُرْتَقَالِ الطَّازِجِ؟<br>1<br>حُصُولِ خَالِدٍ عَلَى الْمَالِ الْكَثِيْرِ كَانَ مِنْ عَمَلِه؟<br>0	.28 رَبْط خَالِد أَسْلاَك الْكَهْرَبَاء كَان خَطَأ كَبِيْرا Potentially GP.<br>.29 كَسْر أَحْمَد قُفْل الْمَحَلّ كَّان لِضَيَاع الْمِفْتَاح Potentially GP.<br>.30 شُرْب عَصِيْر الْبُرْتَقَال الطَّازِج مَمْنُوع عَلَى أَحْمَد Potentially GP.<br>.31 جَمْع خَالِد الْمَال الْكَثِيْر كَان مِن وَالِدَيه Potentially GP<br>.

Set II (RC3: Wrongly vowelized sentences)

Questions/answers	Wrongly vowelized sentences
	Practice sentence (1)
مَنْزِلُ سَعِيْدٌ مَنْزِلٌ صَغِيْرٌ وَبَاعَهُ بِسِعْرٍ رَخِيْصٍ؟<br>0<br>خَالِدٌ اِشْتَرَى لُعْبَةً غَيْرَ مَكْسُورَةٍ؟<br>0<br>الشُّرْبُ وَالأَكْلُ فِيْ الْحَدِيْقَةِ مَمْنُوعٌ؟<br>0<br>أَدَاءُ خَالِدٍ فِيْ مَادَّةِ التَّارِيْخِ كَانَ مُمْتَازَاً؟<br>0<br>جَاءَ فَهْدٌ مِن الْحَدِيْقَةِ مَاشِيَاً عَلَى قَدَمَيْهِ؟<br>0<br>هَلْ أَحْمَدُ يَكْرَهُ أَكْلَ اللَّحْمِ؟<br>1<br>عَدَمُ مَجِيْئِ الْمُوَظَّفِ إِلَى الْعَمَلِ أَدَّى إِلَى فَصْلِهِ؟<br>1<br>الْغُبَاُر كَانَ بِسَبَبِ سُقُوطِ الْمَبْنَى الْكَبِيْرِ ؟<br>1<br>اِسْتَلَمَ مُحَمَّدٌ الرِّسَالَةَ مِن الْمُوَظَّفِ؟	.1 بُاع سُعِيد مُنزُله الكُبَيَر بَسَعَر رُخُيص جُدُا.<br>Wrongly vowelized sentences (2 -9)<br>.2 اَلذِي بُاع خُالُدا اللَعُبُة اِلمِكسَورُة كُان كُاذُبا .<br>.3 إنُ الاِكَل والشَّرُب فُي الُحَدُيقُة غُيُر مُمُنَوع.<br>.4 إُهُمُال خُالُد فُي مُادُة الثُارُيُخ كُان وِاضُحِا.<br>.5 جُاء فِهُد مُن الُحَدُيقُة رِاكِبُا عُلُى دِرُاجُتِه.<br>.6 مُن أِسبِاب مِرُض أُحُمُد عِدَم أِكَله اللُحِم.<br>.7 فِصِل مَدُير الشِرُكُة المَوِظُف لأُنه لُم يِحِضِر.<br>.8 حِصِل غِبُار لَلنِاس بُعُد سِقوط الُمُبَنى الُكُبير.<br>.9 سِلِم مَحُمُد رُسِالة الُمِدُير المِهِمة إِلى المَوِظف.<br><br>Potentially garden-path (10 – 13)<br><br>.10 رِكِض سُعَد فُي صُالُة البِيُت كُان مَزُعِجا Potentially GP.<br>.11 ظِلِم العُامُل فُي الشِركِة كُان بَسِبُب الُمِدُير Potentially GP.

0<br>سَعْدٌ لاَ يَرْكُضُ فِي صَالَةِ الْبَيْتِ؟<br>0<br>الْمُدِيْرُ كَانَ يُعَامِلُ العَامِلَ بِلُطْفٍ وَإِحْسَانٍ؟<br>0<br>سَعْدٌ لاَ يَسْتَطِيْعُ أَنْ يَأكُلَ الأَسْمَاكَ إِذَا أَرَادَ؟<br>1<br>سَعْدٌ مَعْرُوفٌ بِأَمَانَتِهِ؟<br>1	12. أَكِل سُعَد الأَسِماك البُحُرَية غُيَر مُسِمَوح لِه Potentially GP.<br>13. كِسِب سُعَد السَمِعُة الَحِسِنُة كُان بَسُبُب أَمِانُته Potentially GP.

Note: GP= garden-path sentence
Potential GP = if only the consonants of the initial HP-HG word of the sentence are assembled during reading, the sentence should often garden path the reader
