# Supplementary material for: L2 Arabic learners’ processing of Arabic garden-path sentences: a consistent reading pattern
Source: Front Psychol. 2024 Mar 8;15:1333112. doi: 10.3389/fpsyg.2024.1333112 (PMC10957760; doi:10.3389/fpsyg.2024.1333112)
Supplement: Supplementary file 2 [file Table_2.pdf]

Appendix B

English Gloss: examples

Arabic sentence	Arabic Translation	Type of<br><br>Structure
Plain Examplary Sentences		
من أسباب مرض محمد عدم نومه ليلا.<br><br>One of the reasons for Mohammed's illness was his lack of sleep at night.	السَّهَرُ كَانَ مِنْ أسْبَابِ مَرَضِ مُحَمَّدٍ؟<br>1<br><br>The lack of sleep was one of the reasons of Mohammed's illness?	
حدث خوف للسكان بعد حصول الزلزال القوي<br><br>A fear occurred among the residents after the strong earthquake?	خَوْفُ السُّكَّانِ كَانَ بِسَبَبِ صَوَاعِقَ شَدِيْدَةٍ؟<br>0<br>There was fear among the residents after the strong earthquake occurred?	
فتح البائع محله في الصباح كان مبكرا<br><br>The seller opened his shop early in the morning.<br><br>*(Arabic word order: opend/opening + the seller + his + shop + in + the afternoon + was + late.*	البَائِعُ فَتَحَ مَحِلَّهُ فِيْ وَقْتِ الصَّبَاحِ وَلَكِن مُتَأَخِّراً؟<br>0<br>The seller opened his shop in the morning but late?	GP
مرض أحمد بمرض السكري كان لكثرة جلوسه<br><br>Ahmed fell ill with diabetes because he sat a lot.	أحْمَدُ رَجُلٌ سَلِيْمٌ لاَ يَشْتَكِي مِنْ أيِّ أمْرَاضٍ؟<br>0<br>Ahmed is a healthy man who does not suffer from any diseases?	GP
Vowelized-diacritized exemplary sentences		
مِن أسْبَاب مَرَض خَالِد عَدَم أكْله الْخُضْرَوَات.<br><br>One of the reasons for Khaled's illness is that he does not eat vegetables.	خَالِدٌ لاَ يُحِبُّ أكْلَ الْخُضْرَوَاتِ؟<br>1<br>Khaled doesn't like to eat vegetables?	
جَاء صَالِح مِن الْعَمَل مُسْرِعا فِي سَيَّارَته<br><br>Saleh came from work quickly in his	قَادَ صَالِحٌ السَّيَّارَةَ بِسُرْعَةٍ؟<br>1<br><br>Saleh drove his car fast?	

car.		
رَبْط خَالِد أَسْلاَك الْكَهْرَبَاء كَان خَطَأً كَبِيْرا<br><br>Khalid’s connection of the electricity wires was a big mistake?<br><br>*(Arabic word order: connect/connection + Khalid + wires + electricity + was + a + big + mistake.*	خَالِدٌ يَعْرِفُ كَيْفَ يَرْبُطُ أَسْلاَكَ الْكَهْرَبَاءِ؟<br>0<br>Khalid knows how to connect electricity wires?	Potential<br><br>GP
كَسْر أَحْمَد قُفْل الْمَحَلّ كّان لِضَيَاع الْمِفْتَاح<br><br>Ahmed broke the lock of the shop because he lost the key.	لَمْ يَجِدْ أَحْمَدُ مَفَاتِيْحَ الْمَحَلِ, فَقَامَ بِكَسْرِ الْقُفْلِ؟<br>1<br>Ahmed did not find the keys of the store, so he broke the lock?	Potential<br><br>GP
Wrongly vowelized examplary sentences		
مُن إَسبِاب مِرُض أَحُمُد عِدَم إَكَله الْلُحِم<br><br>One of the reasons for Ahmed’s illness is that he does not eat meat.	هَلْ أَحْمَدُ يَكْرَهُ أَكْلَ اللَّحْمِ؟<br>1<br>Does Ahmad hate eating meat?<br>1	
حِصِل غِبُار لَلنِاس بُعُد سِقَوط الْمُبَنى الْكُبير<br><br>People got dust after the big building fell.	الْغُبَار كَانَ بِسَبَبِ سُقُوطِ الْمَبْنَى الْكَبِيْرِ؟<br>1<br>the dust was caused by the collapse of the large building?	
إَكِل سُعَد الإَسِمِاك البُحُرَية غُيَر مُسِمَوح لِه<br><br>Eating sea foods is not allowed for Saad.<br><br>*(Arabic word order: ate/eating + Saad + sea + foods + is + not +allowed + for + him).*	سَعْدٌ لاَ يَسْتَطِيْعُ أَنْ يَأكُلَ الأَسْمَاكَ إِذَا أَرَادَ؟<br>1<br>Saad can not eat fish if he wanted?	Potential<br><br>GP
كِسِب سُعَد السَمِعُة الَحِسِنُة كُان بَسُبُب إَمِانُته<br><br>Saad gained a good reputation because of his honesty.	سَعْدٌ مَعْرُوفٌ بِأَمَانَتِهِ؟<br>1<br>Saad is known for his honesty?	Potential<br><br>GP

Note: the reordering was given for only a GP example from each reading condition.
