# Supplementary material for: L2 Arabic learners’ processing of Arabic garden-path sentences: a consistent reading pattern
Source: Front Psychol. 2024 Mar 8;15:1333112. doi: 10.3389/fpsyg.2024.1333112 (PMC10957760; doi:10.3389/fpsyg.2024.1333112)
Supplement: Supplementary file 3 [file Table_3.pdf]

Appendix C

Reading Aloud Text: version one

وقع حادث سرقة في مدينة بعيدة اسمها "كاتارا"، وكانت السرقة لمجموعة من المحلات في سوق كبير في المدينة. وقد أعلن الخبر في كل الوسائل المرئية والسمعية في المدينة. حدثت السرقة في منطقة تجارية اشتهرت بتجمع المارة للتسوق والتنزه حيث المحلات التجارية وأماكن الترفيه. نتج عن هذه السرقة إصابة حارس الأمن في السوق، وغضب التجار وأصحاب المحلات في هذه المدينة القديمة والمتميزة بوجود أنشطة تجارية عديدة. كشف رجال الشرطة عن موقع السرقة كان غير كافٍ للوصول للسارق. كما أن لجنة التحقيق لم تتوصل لهوية السارق القاتل مع أن أحد شهود العيان قدم وصفاً تقريبياً للسيارة الواقفة بجانب المحل وقت ارتكبت السرقة, والتي انطلقت بطريقة مفاجئة بعد أن سمعت طلقات الرصاص بقليل. وبعد ثلاثة عشر عاما وقعت حادثة سرقة كبيرة جداً في المدينة نفسها، وأعلن الخبر في كل الوسائل المرئية والسمعية في مدينة كاتارا. فتح ملف تلك السرقة والجريمة القديمة بعد حوالي ثلاثة عشر عاماً من وقوعها أعاد للأذهان ذلك النقاش الطويل والصراع المرير الذي عاشته المدينة، وغضب الناس من لجنة التحقيق ازداد، كذلك خوف الناس على أنفسهم وممتلكاتهم عاد من جديد.

ملحوظة: صُمِّم هذا النص لغرض التجربة، والمعلومات الواردة فيه ليست صحيحة

Note: This text was designed for the purpose of experimentation, and the information contained in it is not correct

Reading Aloud Text: verson two

وقع حادث سرقة في مدينة بعيدة اسمها "كاتارا"، وكانت السرقة لمجموعة من المحلات في سوق كبير في المدينة. وقد أعلن الخبر في كل الوسائل المرئية والسمعية في المدينة. حدثت السرقة في منطقة تجارية أُشْتُهِرت بتجمع المارة للتسوق والتنزه حيث المحلات التجارية وأماكن الترفيه. نتج عن هذه السرقة إصابة حارس الأمن في السوق، وغضب التجار وأصحاب المحلات في هذه المدينة القديمة والمتميزة بوجود أنشطة تجارية عديدة. كَشْف رجال الشرطة عن موقع السرقة كان غير كافٍ للوصول للسارق. كما أن لجنة التحقيق لم تتوصل لهوية السارق القاتل مع أن أحد شهود العيان قدم وصفاً تقريبياً للسيارة الواقفة بجانب المحل وقت أُرْتُكِبت السرقة, والتي انطلقت بطريقة مفاجئة بعد أن سمعت طلقات الرصاص بقليل. وبعد ثلاثة عشر عاما وقعت حادثة سرقة كبيرة جداً في المدينة نفسها، وأعلن الخبر في كل الوسائل المرئية والسمعية في مدينة كاتارا. فَتْح ملف تلك السرقة والجريمة القديمة بعد حوالي ثلاثة عشر عاماً من وقوعها أعاد للأذهان ذلك النقاش الطويل والصراع المرير الذي عاشته المدينة، وغَضَبُ الناس من لجنة التحقيق ازداد، كذلك خوف الناس على أنفسهم وممتلكاتهم عاد من جديد.

ملحوظة: صُمِّم هذا النص لغرض التجربة، والمعلومات الواردة فيه ليست صحيحة

Note: This text was designed for the purpose of experimentation, and the information contained in it is not correct
